# Relationship between Upper Limb Functional Assessment and Clinical Tests of Shoulder Mobility and Posture in Individuals Participating in Recreational Strength Training

**DOI:** 10.3390/jcm13041028

**Published:** 2024-02-10

**Authors:** Magdalena Zawadka, Marta Gaweł, Agnieszka Tomczyk-Warunek, Karolina Turżańska, Tomasz Blicharski

**Affiliations:** 1 Department of Sports Medicine, Chair of Clinical Physiotherapy, Faculty of Health Sciences, Medical University of Lublin, Chodzki 15, 20-093 Lublin, Poland; magdalena.zawadka@umlub.pl (M.Z.); reh-ortop@umlub.pl (M.G.); 2 Laboratory of Locomotor Systems Research, Department of Rehabilitation and Physiotherapy, Medical University of Lublin, Jaczewskiego 8, 20-954 Lublin, Poland; 3 Department of Rehabilitation and Orthopaedics, Medical University of Lublin, Jaczewskiego 8, 20-954 Lublin, Poland; tomasz.blicharski@umlub.pl

**Keywords:** muscle, strength training, upper limb, closed kinetic chain

## Abstract

Background: The upper limb is crucial for functioning in everyday life, thus comprehensive assessment is crucial for physically active people to monitor the effect of exercise and prevent injuries. The aim of this study was to analyse the relationship between upper limb function, shoulder mobility, and posture in individuals who participate in recreational strength training. Methods: Thirty-four subjects who engaged in strength training of the upper limbs were divided into two groups: Group 1 (exercise < 3 years) and Group 2 (exercise ≥ 3 years). Lateral scapular slide tests, head and clavicle posture evaluations, and shoulder mobility and closed kinetic chain tests were performed. Results: Group 1 had a greater flexion deficit in both shoulders than Group 2. There was greater external rotation in the non-dominant shoulder and a greater score of the closed kinetic chain test in Group 2 compared to Group 1. There were no statistically significant differences between groups regarding scapula, clavicle, and head posture. The closed kinetic chain test was correlated with a scapula position and symmetry in shoulder flexion in Group 2. Conclusions: Long-term strength training of the upper limbs can be recommended to improve functional abilities in the closed kinetic chain, increase shoulder mobility, and reduce asymmetry.

## 1. Introduction

The upper limb is crucial for functioning in everyday life, mainly due to its anatomical structure. Thanks to this, we can perform complex movements, including lifting, catching, and turning over objects. It is also used for communication and conveying emotions, thus contributing to maintaining interpersonal interactions [1].

However, the specific anatomical structure of the upper limb contributes to an increased risk of numerous and frequent injuries. Epidemiological data show that upper limb injuries are among the most common injuries in sports and other physical activities [2,3]. Epidemiological studies have shown that from 20 to 40% of all injuries reported to the emergency department are injuries to the upper limb [4]. In cases of people practising sports or engaging in other physical activities, shoulder injuries occur the most often [3]. It has been shown that shoulder injuries constitute 8 to 13% of all sports injuries [2]. Regardless of the type and location of the injury, they must be diagnosed as soon as possible so that the athlete or person exercising recreationally can return to physical activity as soon as possible [3].

The risk of injury is related to physical fitness. The better the person’s physical fitness, the lower the risk of injury. It is associated with maintaining balance and increasing muscle strength, which correlates with neuromuscular control and proper sensorimotor integrity [5,6,7]. Physical fitness can be assessed using various tests, including functional tests like the closed kinetic chain upper limb test, mobility tests, and tests related to upper body posture. All these tests are used to assess the fitness of the upper limb. However, some authors point out that they are only partially reliable because their results depend on the position of the scapula and shoulder asymmetry, which distorts the results [6]. However, they are used in practice because, currently, there are no other methods of assessing fitness that are so readily available, non-invasive, and inexpensive [8,9]. Therefore, newer and more reliable tests are being sought, as well as the relationship between complex tests and individual measurement parameters [10]. Despite the importance of this issue, the relationship between upper limb physical performance tests has not been widely studied.

One of the factors that may reduce the risk of upper limb injuries and their long-term consequences may be appropriately performed recreational training. The available literature lacks research results regarding the impact of reaction training on the efficiency of the upper limb. However, literature data indicate that the use of additional physical activity during free time in patients suffering from low back pain influences the prognosis of these patients [11].

This study has two objectives: first, to determine posture/function differences among participants with different training durations and, second, to analyse correlations between measurement parameters. We hypothesize that strength training is associated with the functional performance of the upper limb, shoulder mobility, and posture.

## 2. Materials and Methods

### 2.1. Subjects

**Figure 1:**
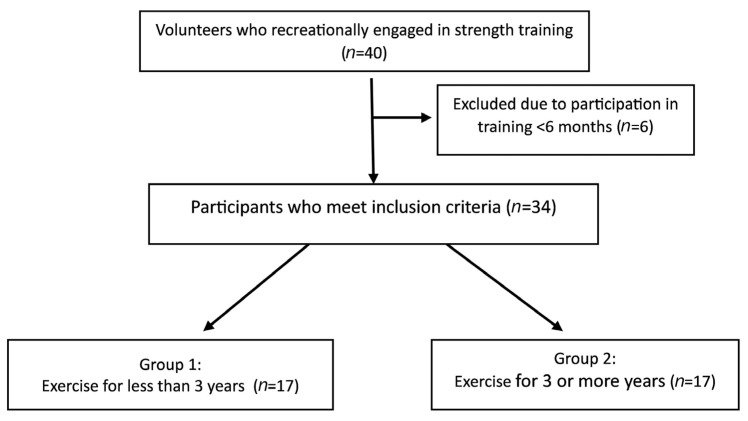
Flowchart diagram: recruitment of participants in the study.

The research material consisted of a sample of 34 subjects ages 22–40 who recreationally engaged in strength training (Figure 1). Participants did not take part in sports competitions, including amateur competitions. The study was conducted between November 2022 and January 2023 on clients of the training centre, using a cross-sectional design. The inclusion criteria were an engagement in strength exercise with external resistance at least once a week for more than 6 months. The participants were divided into two groups, with a balance of male and female participants, based on the criteria used in a previous study [12]: beginners who exercised for less than 3 years (*n* = 17, 10 females and 7 males) and experienced who participated for 3 or more years in upper limb exercise (*n* = 17, 9 females and 8 males). There were no statistically significant differences in the number of males and females in both groups (chi^2^ = 0.12, *p* = 0.73). The sample size of a minimal number of 30 volunteers was determined based on a previous study [7].

People who reported pain or feeling unwell during the tests, pregnant women, and people who did not consent to participate in the study were excluded from participation in the study. Informed consent and study protocol were approved by the bioethics committee (no. KE-0254/233/11/2022). The study was conducted following the Declaration of Helsinki.

### 2.2. Procedures

Participation in strength training applies to exercises using body weight (callisthenics) and exercises with external resistance (dumbbells, barbells, elastic bands). The study participants declared that they undertook each type of training at least once a week. The external resistance training was performed under the supervision of a trainer in the training centre. The load was individually selected, taking into account training experience and strength capabilities. The weight of the barbell and dumbbell was about 80% of repetition maximum (1RM), and the pace was slow. Participants could increase the weight by 1 unit if they reached their maximum number of repetitions.

**Figure 2:**
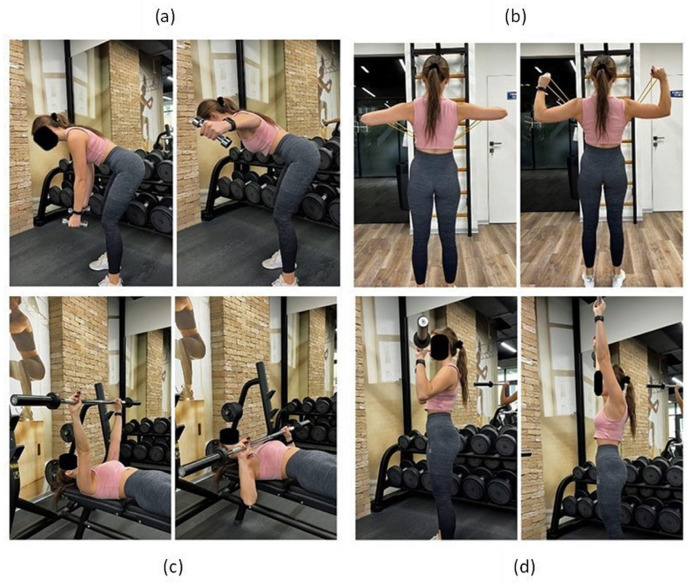
Execution of exercises: (**a**) bend over dumbbell row; (**b**) face pull; (**c**) chest press; (**d**) military press. Description in the text.

Training included four exercises (Figure 2):

(a) Bend over dumbbell row: an exercise involving the deltoid muscle, latissimus dorsi rhomboids and trapezius [13]. The exercise is performed in 3 sets of 10–15 repetitions. Dumbbell lifting movement is initiated by external rotation of the shoulders and scapula retraction.
(b) Face pull: an exercise aimed at engaging the posterior deltoid, teres minor, trapezius, rhomboid major, and infraspinatus muscles [14]. The exercise is performed in 3 sets of 10–15 repetitions. External rotation movement is initiated by retraction and depression of the scapulae.
(c) Chest press: an exercise involving mainly the pectoralis major muscle. The exercise is performed in 3 sets of 5–10 repetitions of lowering the barbell to the chest.
(d) Military press: an exercise involving anterior and middle supraspinatus muscle and the pectoralis major muscle. The exercise is performed in 3 sets of 8–12 repetitions. The barbell is pressed upwards, with retraction of the head.

### 2.3. Lateral Scapular Slide Test

The test tool was a centimetre tape. Measurements were made between the medial edge of the scapula and the spine in three positions of the upper limbs in the resting position, with hands on the hips and with the arm abducted to 90 degrees and in internal rotation. The test was performed by measuring the distance of the scapula crest and lower angle of the scapula from the nearest spinous process of the thoracic spine in resting position and then in two other positions relative to the same spinous process [15,16]. Each measurement was repeated twice with a minimum break of 45 s. The absolute difference between dominant (dom) and non-dominant limbs (ndom) was also calculated to determinate asymmetry. The dominance for the upper limb was established by the answer to the following question: which hand do you use for writing?

### 2.4. Assessment of Clavicle Alignment

**Figure 3:**
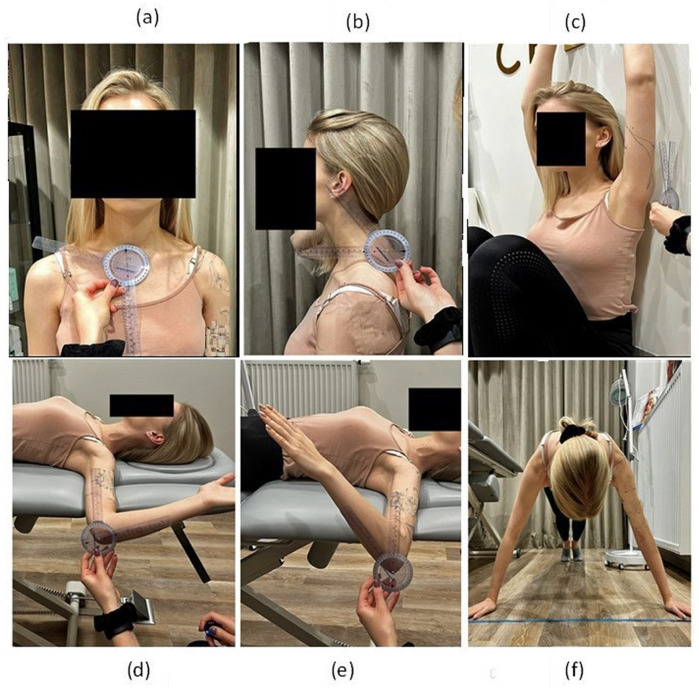
Measurements: (**a**) clavicle’s position; (**b**) craniovertebral angle; (**c**) back-to-wall shoulder flexion test; (**d**) external rotation of the arm; (**e**) internal rotation of arm; (**f**) closed kinetic chain upper limb test.

Assessment of the clavicle’s position about the long axis of the sternum was measured with a standard plastic goniometer (Figure 3). The examined subject was standing in a resting position, and the angle between the long axis of the sternum and the clavicle was measured using a goniometer on the right and left sides [17,18]. The measurement was performed twice with a break of at least 45 s.

### 2.5. Assessment of Head Position

Measurement of the craniovertebral angle was used for assessing head posture (Figure 3). This is the angle formed by a horizontal line drawn through the spinous process of the seventh cervical vertebra (C7) and the line connecting the spinous process of the C7 vertebra with the tragus of the ear [19]. The angle was measured using a goniometer twice in the subject’s free-standing and sitting positions.

### 2.6. Shoulder Mobility: Back-to-Wall Shoulder Flexion Test

Before taking the measurements, the subjects could practice each movement 3–5 times. A back-to-wall shoulder flexion test allowed for the measurement of the angle of flexion deficit in the shoulder joint in a sitting position with the back resting against the wall and the hip joints maximally bent (Figure 3). The test eliminates the possibility of compensating for flexion deficits in the shoulder joint by preventing trunk movements. The angle between the wall and the lateral epicondyle of the humerus on the right and left sides was measured with a goniometer. The measurement was performed twice with a break of at least 45 s.

### 2.7. Shoulder Mobility: Rotations

The angle of external and internal rotation was measured in the supine position with the arm abducted up to 90 degrees (Figure 3). The subject’s task was to perform maximum external and internal rotation (while holding the scapula in contact with the table) in the shoulder joint. The rotation angle was measured using a goniometer on the right and left sides. The measurement was performed twice with a break of at least 45 s.

### 2.8. Closed Kinetic Chain Upper Limb Test

The upper limb test in a closed kinematic chain involves measuring the number of taps on the tape within 15 s. The starting position is front support (push-up position) with the hands 36 inches apart (Figure 3). The subject’s task was to make as many touches as possible on the opposite hand (alternately) within 15 s [20,21]. The number of touches was counted, and the subject had two trials 45 s apart.

### 2.9. Statistical Analysis

To perform statistical analyses, Statistica software (Tibco, version 14.00) was used. Compliance with normal distribution was assessed using the Shapiro–Wilk test. To compare body postures/functions between strength trainees with different training durations (Groups 1 and 2), we employed the Mann–Whitney U test. Effect size (ES) of Mann–Whitney U test was interpreted as small (0.1–0.3), moderate (0.3–0.5), and large (>0.5) [22]. The relationship between the closed kinetic chain test and other test results was analysed using Spearman’s rank correlation coefficient. We interpreted the correlation as very strong (r > 0.70), strong (r = 0.40–0.69), moderate (r = 30–39), weak (r = 20–29) or insignificant (r < 20). The assumed level of statistical significance was *p* < 0.05.

To determine the influence of individual variables on the determination of belonging to the beginners (0) or experienced (1) groups, a linear regression analysis was performed. It was assumed that all variables were included in the model, therefore tests of significance were carried out based on the likelihood ratio (LR). The LR test is a test of the sufficiency of a smaller model compared to a more complex model. There was no linear impact of the studied variables. There were also no statistically significant interactions between variables that should be included in the model. Additionally, following the rules indicating the minimum sample size for the number of variables included in the regression model, their number was reduced from those indicated as significant, and the sex variable was also taken into account. The model was built based on all effects and then limited by excluding data with the least impact until a model was obtained that maximally explained the studied phenomenon (maximum R^2^ and minimum standardised error; SE).

The sample size of a minimal number of 30 volunteers was determined based on a previous study [7]. The sample size required to achieve a power of 0.80 with a significance level of 0.05 and a correlation coefficient outside the interval [−0.50 to 0.50] was 16. An analysis of power was conducted using G*Power 3.1 [23].

## 3. Results

There were no statistically significant differences between the groups in terms of age, height, body mass, and BMI. Detailed results are presented in Table 1.

There were no statistically significant differences between the groups in terms of scapula and clavicle position or head posture. Detailed results are presented in Table 2.

There were statistically significant differences between the groups in terms of the back-to-wall shoulder test. Group 1 had a greater deficit of shoulder flexion in both dominant (*p* < 0.001, ES = 0.58, large effect size) and non-dominant (*p* = 0.002, ES = 0.52, large effect size) arms and a greater difference between sides (*p* = 0.04, ES = 0.35, moderate effect size) than Group 2. There was also statistically significant greater external rotation in the non-dominant shoulder (*p* = 0.047, ES = 0.34, moderate effect size) in Group 2 compared to Group 1. Group 2 had significantly greater scores of the closed kinetic chain test (*p* = 0.03, ES = 0.37, moderate effect size) than Group 1 (Table 3).

There were only two statistically significant correlations between the closed kinetic chain test and other parameters in Group 1 (Table 4). Results of the closed kinetic chain test was strongly correlated with a lower angle position on the dominant side during shoulder abduction (r = 0.51, *p* = 0.04). There was also a strong positive correlation between the closed kinetic chain test and external rotation on the non-dominant side (r = 0.50, *p* = 0.04).

Results of the closed kinetic chain test was strongly correlated with a scapula crest position on the dominant and non-dominant side in all positions (dominant-rest: r = 0.56, *p* = 0.02; dominant-hips: r = 0.67, *p* < 0.01; dominant-abduction: r = 0.61, *p* = 0.01; non-dominant-rest: r = 0.68, *p* < 0.01; non-dominant-hips: r = 0.67, *p* < 0.01; non-dominant-abduction: r = 0.49, *p* = 0.048). Results of the closed kinetic chain test was strongly negatively correlated to the difference of the back-to-wall shoulder test between the sides (r = −0.52, *p* = 0.03).

Based on the linear regression analysis, the estimated model explains approximately 60% of the examined relationship. The average standard error of the estimate is 34%. The slope coefficient for the sex variable was statistically insignificant, which means that the regression lines for both sexes are not statistically different. The back-to-wall shoulder test (on the non-dominant side, β = −0.67, *p* < 0.001), internal rotation (on the dominant side, β = −0.38, *p* < 0.01), and the closed kinetic chain test (β = 0.40, *p* = 0.03) were included in this model. These parameters were statistically related to the duration of training (Table 5).

## 4. Discussion

This study examined how recreational strength training affects upper limb function, shoulder mobility, and posture. Current research on prolonged recreational strength training has revealed some interesting findings. One of the key findings is that it leads to an increase in external rotation of the shoulder. Additionally, it improves the uncompensated flexion of the shoulder (as measured by the back-to-wall shoulder test) and enhances functional abilities in the closed kinetic chain. The findings provide some evidence supporting the hypothesis, but further research may be necessary to confirm its validity fully.

Physical activity (PA) positively affects the entire human body by influencing both mental and physiological health. It contributes to maintaining physical fitness and positively affects the efficiency and condition of the body, thus reducing the risk of chronic diseases of the locomotor system, respiratory system, and circulatory system and ailments such as diabetes, hypertension, and obesity [24,25]. Maintaining PA also reduces the risk of limb injuries [5,6,7,26].

Upper limb injuries are one of the most common injuries in sports. Epidemiological data show that they affect 8 to 13% of athletes/physically active people [2], constituting 20–40% of injuries reported to emergency departments [4]. However, injuries and/or dysfunctions of the upper limbs are not only related to accidents or physical activity. Still, they may also occur due to work (WRMD—work-related musculoskeletal disorders) [27,28]. WRMDs are a group of painful conditions that affect the human body’s muscles, tendons, ligaments, nerves, and joints. These disorders are typically caused by prolonged exposure to physical exertion, repetitive motion, and body postures in the workplace. They can also be influenced by psychosocial factors such as stress and poor working conditions [28]. It is important to identify and address the risk factors associated with WRMDs in the workplace to prevent their occurrence and reduce their impact. This can be achieved through ergonomic assessments, training programs, job redesign, and workplace modifications. By promoting a safe and healthy work environment, employers can help minimize the risk of WRMDs and improve the well-being of their employees. Research has shown that not only adapting the workplace to the type of work performed has a beneficial effect on reducing the risk of WRMD but also performing additional exercises reduces its frequency, especially with the use of strength training, which positively affects the body, reduces pain, improves performance, increases strength, and rebuilds muscle tissue [28,29]. Research carried out on various professional groups has shown that the introduction of appropriate, individually tailored strength training has a positive effect on the reduction of pain and improves the efficiency of the upper limbs [30,31].

The prolonged duration of the injury contributes to a decrease in the patient’s physical fitness, negatively affecting the muscle mass and causing its atrophy [32]. Studies assessing the impact of physical training show that it improves the strength of the upper limbs, which positively affects the patient’s fitness and ability to perform ADLs (activities of daily living) [33].

Studies have shown that strength training has a positive effect on muscle strength, which has a significant impact on ADLs, thus reducing the risk of disability associated with diseases of the locomotor system and circulatory system [33,34]. Strength training is also used in patient rehabilitation. In the study by Perret et al. (2022), adding 12 weeks of strength training during the standard rehabilitation process in stroke patients contributed to an increase in muscle strength, and similar results were observed in a clinical study conducted by Jacobs et al. 2001 in which resistance training was also used for 12 weeks [35,36]. Strength training, as a form of rehabilitation treatment, is also used in patients after spinal cord injury who experienced partial paralysis or secondary weakness. A study by Bye et al. (2016) showed that using strength training in patients after spinal cord injury significantly improved muscle strength in partially paralyzed muscles [37].

The available literature lacks studies that assess how the use of recreational strength training affects the fitness of the upper limbs. However, as previously described, strength training significantly improves strength. It reduces pain in patients suffering from various diseases [30,31,35,36,37]. However, it should be noted that none of these studies examined the function of the upper limbs using fitness tests. Therefore, our study aimed to assess whether recreational strength training improves the function of the upper limbs.

Our study showed that long-term strength training of the upper limbs increases the range of motion and improves the strength and support functions of the upper limbs. Previous studies indicated that strength training and stretching interventions were not different in their effects on range of motion [38]. Incorporating full-range resistance training into a workout routine can enhance flexibility as effectively as stretching [39]. The findings of Barlow et al. (2002) indicate that bodybuilders exhibit a decreased range of motion in shoulder rotation compared to the control group [40]. However, recreational resistance training can positively impact maintaining or enhancing mobility [41]. Therefore, it may be recommended to incorporate recreational resistance training into one’s fitness routine to improve strength and mobility. The regression results in our study indicated that the duration of training is related to functional abilities in the closed kinetic chain, increased shoulder flexion in the back-to-wall test, and limitation of the internal rotation of the shoulder. Limitation of the internal rotation of the shoulder is often related to the adaptation to external rotation movements performed in training. This situation is commonly observed in many overhead-throwing sports and does not necessarily mean pathology [42]. This aspect of recreational training requires further research.

Our study observed no relationship with body posture, which is likely explained by adaptive changes in less than three years of training. Previous research indicates that several weeks of strength training is sufficient to change body posture [43,44]. That is probably a reason why our study found no differences between the groups in body posture.

The closed kinetic chain upper limb is a test that allows quantitative assessment of the efficiency of the upper limbs. It is easy to perform and does not require specialized equipment. In previous literature, the reliability of this test has been investigated due to its significant dependence on sex., physical activity, shoulder disjunction, and pain [45]. In the current study, the groups were balanced by sex. Statistical analysis showed no significant differences between the groups in terms of age, height, body mass, and BMI. The differences in physical activity are that one group of participants had been training for less than three years, and the other had been training for more than three years. Statistical analysis showed that Group 2 scored significantly more on the closed kinetic chain test than Group 1. Correlations were also observed between the closed kinetic chain upper limb test and scapular positioning, which may result from the development of the muscles stabilizing the scapula due to strength training. However, other research suggests that muscle strength is unrelated to scapular position. It was found that no significant association exists between the positioning of the scapula and the force exerted by the middle trapezius or pectoralis minor muscles [46]. However, the current study position of the scapula has been found to correlate with the functional performance abilities displayed during exercises operating in a closed kinetic chain.

## 5. Study Limitation

The main limitation of our study is the small group of patients. However, most of the studies examining the effects of resistance training on upper limb injuries/dysfunctions have been conducted in small groups of patients [28,30,31,35]. Our study did not assess specific muscle strength but rather focused on functional abilities of the participants. Our research is the first to evaluate how recreational strength training affects upper limb performance, opening a new direction for research. Another strength of our work is that the statistical analysis did not show significant differences between the study groups in terms of age, weight, height, and BMI. Therefore, these parameters did not influence differences in fitness tests.

## 6. Conclusions

The results of our study are of great importance to practitioners of sports medicine, kinesiology, and physiotherapy. They showed that long-term strength training of the upper limbs is associated with improved stability and strength in assessing the closed kinetic chain, increased shoulder mobility, and reduced interlateral asymmetry. Upper limb closed kinetic chain performance is related to the scapular position in people participating in long-term recreational strength training. Our work provides a better understanding of the factors contributing to optimal performance and injury prevention in recreational strength training, which will aid in developing evidence-based interventions to improve upper extremity function, shoulder mobility, and posture. However, conclusions regarding strength training should be drawn with caution due to the small sample size and observational design of the study.

## Figures and Tables

**Table 1:** Participants’ characteristics.

Variable	Group	Mean	Std. Dev.	Median	Min–Max	Statistics	
						Z	*p*
Age [years]	Beginner	27.53	6.02	25	22–40	−1.69	0.09
	Experienced	30.12	5.01	29	22–39		
Heigh [cm]	Beginner	175.59	8.92	176	159–193	0.33	0.74
	Experienced	175.06	9.66	173	162–193		
Body mass [kg]	Beginner	69.47	11.54	68	50–90	−0.26	0.80
	Experienced	72.24	16.36	65	50–98		
BMI [kg/m^2^]	Beginner	22.46	2.85	21.56	18.82–28.08	−0.84	0.40
	Experienced	23.26	2.95	22.86	18.82–27.78		

Std. Dev—standard deviation; Z-score of the Mann–Whitney test.

**Table 2:** Results obtained in measurements of scapula, clavicle, and head position.

Variable	Group	Mean	Std. Dev.	Median	Min	Max	Statistics	
							Z	*p*
Scapula crest-dom-rest [cm]	Beginner	5.90	1.09	5.75	4.50	8.00	−1.42	0.16
	Experienced	6.50	1.22	6.50	4.75	9.00		
Scapula crest-dom-hips [cm]	Beginner	5.34	1.19	5.00	3.75	8.00	−0.83	0.41
	Experienced	5.65	1.18	5.50	3.50	7.75		
Scapula crest-dom-abduction [cm]	Beginner	3.87	0.90	4.00	2.50	6.00	−0.76	0.45
	Experienced	4.29	1.43	4.25	2.00	6.75		
Scapula crest-ndom-rest [cm]	Beginner	5.68	1.23	5.50	4.00	8.00	−1.09	0.28
	Experienced	6.19	1.24	6.00	4.00	8.50		
Scapula crest-ndom-hips [cm]	Beginner	5.07	1.16	5.00	3.50	7.00	−0.92	0.36
	Experienced	5.43	0.84	5.25	4.00	7.00		
Scapula crest-ndom-abduction [cm]	Beginner	4.00	1.54	4.00	2.50	9.00	−0.40	0.69
	Experienced	4.09	1.31	4.00	2.00	6.25		
Scapula crest—Difference -rest [cm]	Beginner	0.75	0.49	0.75	0.00	2.00	0.30	0.77
	Experienced	0.78	0.71	0.50	0.00	3.00		
Scapula crest—Difference -hips [cm]	Beginner	0.79	0.64	0.50	0.00	2.50	0.97	0.33
	Experienced	0.51	0.30	0.50	0.00	1.00		
Scapula crest—Difference -abduction [cm]	Beginner	0.54	0.75	0.25	0.00	3.00	−0.56	0.57
	Experienced	0.47	0.32	0.50	0.00	1.00		
Lower angle-dom-rest [cm]	Beginner	8.76	1.40	9.00	6.00	11.00	0.71	0.48
	Experienced	8.44	1.37	8.50	6.50	10.50		
Lower angle-dom-hips [cm]	Beginner	8.96	1.57	8.75	7.00	12.50	−0.05	0.96
	Experienced	8.79	1.35	8.50	6.00	11.00		
Lower angle-dom-abduction [cm]	Beginner	8.87	1.67	9.00	6.00	12.00	−0.26	0.79
	Experienced	8.96	1.80	9.00	5.00	12.25		
Lower angle-ndom-rest [cm]	Beginner	8.15	1.32	8.00	5.50	10.75	−0.14	0.89
	Experienced	8.35	1.36	8.00	6.00	11.00		
Lower angle-ndom-hips [cm]	Beginner	8.60	1.49	8.25	6.50	11.50	−0.36	0.72
	Experienced	8.71	1.48	8.50	6.50	12.00		
Lower angle-ndom-abduction [cm]	Beginner	9.09	1.43	9.00	6.50	11.00	0.26	0.80
	Experienced	8.87	1.90	9.00	5.00	11.75		
Lower angle- Difference -rest [cm]	Beginner	0.97	0.86	0.75	0.25	3.50	1.35	0.18
	Experienced	0.59	0.38	0.50	0.00	1.50		
Lower angle- Difference -hips [cm]	Beginner	0.97	0.86	0.50	0.00	3.00	1.12	0.26
	Experienced	0.59	0.45	0.50	0.00	1.50		
Lower angle- Difference -abduction [cm]	Beginner	1.04	1.02	1.00	0.00	3.00	0.19	0.85
	Experienced	0.79	0.84	0.50	0.00	2.75		
Head posture in standing [°]	Beginner	47.35	5.98	48.00	36.00	59.00	−1.42	0.16
	Experienced	50.26	4.80	49.00	40.00	61.50		
Head posture in sitting [°]	Beginner	46.71	7.00	47.00	36.00	60.00	−1.12	0.26
	Experienced	48.71	6.02	50.00	38.00	65.00		
Clavicle position dom [°]	Beginner	110.03	6.06	110.00	99.00	120.50	1.95	0.05
	Experienced	105.50	6.09	105.00	95.00	119.00		
Clavicle position ndom [°]	Beginner	109.35	4.38	110.00	101.00	116.00	0.97	0.33
	Experienced	107.38	5.38	106.00	99.50	116.00		
Clavicle difference [°]	Beginner	3.38	3.09	2.00	0.50	11.00	−0.33	0.74
	Experienced	4.00	4.30	3.00	0.00	17.50		

Dom—dominant side; ndom—non-dominant side; std. dev—standard deviation; Z-score of the Mann–Whitney test.

**Table 3:** Results of functional and mobility tests.

Variable	Group	Mean	Std. Dev.	Median	Min	Max	Statistics	
							Z	*p*
Back-to-wall shoulder test dom [°]	Beginner	19.71	12.39	20.00	0.00	50.00	3.37	<0.001 * ES = 0.58 (large)
	Experienced	6.41	6.64	5.00	0.00	20.00		
Back-to-wall shoulder test ndom [°]	Beginner	18.65	12.95	20.00	0.00	46.00	3.06	0.002 * ES = 0.52 (large)
	Experienced	5.59	5.39	5.00	0.00	14.00		
Back-to-wall shoulder test diff [°]	Beginner	6.00	5.41	4.50	0.00	20.00	2.06	0.04 * ES = 0.35 (moderate)
	Experienced	2.76	2.88	2.00	0.00	9.50		
Internal rotation dom [°]	Beginner	57.15	11.98	53.00	37.50	76.50	0.95	0.34
	Experienced	52.24	12.45	51.00	29.50	72.00		
Internal rotation ndom [°]	Beginner	60.65	10.63	60.50	40.00	76.00	1.29	0.20
	Experienced	55.82	11.75	57.00	35.50	75.00		
Internal rotation difference [°]	Beginner	6.79	7.68	5.00	1.00	35.00	−0.90	0.37
	Experienced	8.47	6.73	5.00	2.50	24.00		
External rotation dom [°]	Beginner	86.32	10.10	85.00	70.00	105.00	−1.74	0.08
	Experienced	94.38	13.84	94.50	71.50	131.50		
External rotation ndom [°]	Beginner	87.53	12.06	87.00	65.00	115.00	−1.98	0.047 * ES = 0.34 (moderate)
	Experienced	94.76	10.91	95.00	72.00	116.00		
External rotation difference [°]	Beginner	6.68	5.74	6.00	0.00	21.00	−0.03	0.97
	Experienced	6.50	4.61	5.00	1.50	15.50		
Closed kinetic chain test [repetitions}	Beginner	22.12	2.78	22.00	18.50	30.00	−2.16	0.03 * ES = 0.37 (moderate)
	Experienced	25.65	5.10	24.00	19.00	35.00		

*—significant difference *p* < 0.05; std. dev—standard deviation; Z-score of the Mann–Whitney test; ES-effect size.

**Table 4:** Correlation between closed kinetic chain test and physical performance tests in both groups.

Variable	Group 1 (Beginner)		Group 2	
	Spearman *r*	*p*-Value	Spearman *r*	*p*-Value
Scapula crest-dom-rest	0.31	0.22	0.56 (strong)	0.02 *
Scapula crest-dom-hips	0.08	0.75	0.67 (strong)	<0.01 *
Scapula crest-dom-abduction	−0.05	0.86	0.61 (strong)	0.01 *
Scapula crest-ndom-rest	0.42	0.09	0.68 (strong)	<0.01 *
Scapula crest-ndom-hips	0.41	0.10	0.67 (strong)	<0.01 *
Scapula crest-ndom-abduction	0.30	0.24	0.49 (strong)	0.048 *
Lower angle-dom-rest	0.48	0.05	0.27	0.29
Lower angle-dom-hips	0.32	0.22	0.28	0.27
Lower angle-dom-abduction	0.51 (strong)	0.04 *	0.30	0.25
Lower angle-ndom-rest	0.33	0.19	0.46	0.06
Lower angle-ndom-hips	0.38	0.13	0.27	0.29
Lower angle-ndom-abduction	0.34	0.18	0.24	0.35
Scapula crest—Difference 1	0.30	0.24	−0.14	0.58
Scapula crest—Difference 2	0.22	0.39	0.02	0.94
Scapula crest—Difference 3	−0.07	0.80	−0.13	0.62
Lower angle- Difference 1	0.21	0.41	0.03	0.91
Lower angle- Difference 2	−0.16	0.54	0.21	0.43
Lower angle- Difference 3	0.10	0.70	0.08	0.75
Head posture in standing	−0.13	0.61	−0.04	0.89
Head posture in sitting	−0.11	0.67	0.08	0.77
Clavicle position dom	0.07	0.79	0.23	0.37
Clavicle position ndom	0.06	0.83	−0.08	0.76
Clavicle difference	0.05	0.84	−0.01	0.96
Back-to-wall shoulder test dom	0.26	0.32	0.13	0.63
Back-to-wall shoulder test ndom	0.28	0.27	0.23	0.38
Back-to-wall shoulder test difference	−0.25	0.34	−0.52 (strong)	0.03 *
Internal rotation dom	−0.33	0.19	−0.23	0.38
Internal rotation ndom	−0.18	0.48	−0.07	0.78
Internal rotation difference	−0.09	0.73	0.04	0.89
External rotation dom	0.30	0.24	−0.22	0.40
External rotation ndom	0.50 (strong)	0.04*	−0.38	0.13
External rotation difference	0.14	0.58	−0.36	0.15

*—significant difference *p* < 0.05; std. dev—standard deviation; Z-score of the Mann–Whitney test.

**Table 5:** Regression summary.

Variables	β	SE β	B	SE B	t	*p*-Value	Statistics
Intercept	-	-	18.55	17.49	1.06	0.29	R = 0.78 R^2^ = 0.60 SE = 0.34
Sex	−0.18	0.17	−0.17	0.17	−1.00	0.32	
Back-to-wall shoulder test ndom	−0.67	0.13	−0.03	0.006	−4.92	<0.001 *	
Internal rotation dom	−0.38	0.13	−0.02	0.005	−2.83	<0.01 *	
Closed kinetic chain test	0.40	0.17	0.04	0.02	2.27	0.03 *	

*—significant difference *p* < 0.05; SE—standardized error; B—non-standardised coefficient value; R—multiple correlation coefficient; R square—coefficient of determination.

## Data Availability

The data presented in this study are available from the author upon request.
